# Cognitive Enhancement: Perceptions Among Parents of Children with Disabilities

**DOI:** 10.1007/s12152-014-9201-8

**Published:** 2014-02-21

**Authors:** Natalie Ball, Gregor Wolbring

**Affiliations:** Community Rehabilitation and Disability Studies, Department of Community Health Sciences, Faculty of Medicine, University of Calgary, Calgary, Alberta Canada

**Keywords:** Cognitive enhancement, Parents, Children with disabilities, Ethics

## Abstract

Cognitive enhancement is an increasingly discussed topic and policy suggestions have been put forward. We present here empirical data of views of parents of children with and without cognitive disabilities. Analysis of the interviews revealed six primary overarching themes: meanings of health and treatment; the role of medicine; harm; the ‘good’ parent; normality and self-perception; and ability. Interestingly none of the parents used the term ethics and only one parent used the term moral twice.

## Introduction

Cognitive enhancement (CE) has sparked controversy within academia and the media in recent years [1–4]. Various definitions of cognitive enhancement (CE) exist within the literature [5, 6]. For the purposes of this study, the researchers chose to employ CE as the use of interventions to improve cognitive functioning *above* a level that is considered to be ‘normal’ or species typical for humans. Defining CE as improving cognitive functioning above a species-typical level accounts for shifting ability norms and allows for a broader exploration of potential CE interventions. A myriad of ethical and philosophical issues pertaining to CE’s have been identified—the safety and efficacy of these products as well as how they affect personal identity and authenticity to name only a few areas of contention [4, 7]. Despite claims of widespread use and effective means to achieve CE and despite that policy suggestions have already been put forward [8–10], evidence and knowledge pertaining to CE’s are extremely limited at this time and numerous authors have acknowledged the need for further research [2, 9, 11, 12].

There has been little evidence collected about how CE’s are perceived within and between various groups [3, 4]. The few studies that have been conducted on CE perception have identified complex considerations like the effects of CE on identity, societal expectations and fairness, but these conceptions seem to qualitatively differ based on the group that is being researched [9, 13, 14]. For example, Forlini and Racine [7] conducted a qualitative study questioning groups of university students, parents of university students, and health care providers what their perceptions of using methylphenidate as CE’s are. They identified that some of the issues underlying their perceptions of CE included authenticity, specifically whether or not the achievements you make while using CE’s can be considered ‘real’; fairness relating to honesty and equality; and external factors, such as legality and societal perception. Further, these authors suggested that societal portrayals and regulations of CE’s influence how they are perceived as aligning with personal value systems. Alternatively, studies by Banjo et al. [13] and Mendelsohn [15] showed that while physicians expressed those concerns which have been identified by other groups of individuals, they tended to focus on those issues which were at odds with their role as a physician. This suggests that the role an individual occupies may influence which factors are seen as most important with respect to CE use, and that it may be fruitful to explore how parent’s perceptions of CE are influenced by what they think the role of a parent entails and what expectations society has of them in order to be a successful parent. Furthermore, disabled people and various groups linked to disabled people from parents to staff of disability service organizations to professionals working with disabled people to teachers are underrepresented in the CE discourse. Because the long-term social implications of various CE’s are unknown, but the portrayal of these products has been overwhelmingly positive within the media, it is crucial to understand what factors will affect whether or not individuals will want to take CE’s or encourage their use for others [4, 9, 16]. Therefore, in-depth exploration from a variety of different perspectives seems warranted. Our study will help to address the need to further our understanding of CE perception from various perspectives by conducting an exploration with two specific groups—parents of young (3–9 year old) nondisabled children and parents of dependent, cognitively disabled children.

Though there are limited data pertaining to parents and CE perception, literature on parent perceptions of medication use in children with attention deficit hyperactivity disorder (ADHD) provided a useful starting point for this study. In a qualitative study conducted by Singh (2005), some parents justified the use of medication for their child because they felt that the drugs were facilitating the expression of their child’s identity, not changing it [17]. When a behaviour associated with ADHD went against a culturally valued enterprise, like social integration or success in school, parents were less likely to see this as a part of their child’s identity and were more likely to accept that trait as pathological. This study raised interesting questions about ability, pathology and acceptability of interventions that will be useful for this project. Because parents were more willing to accept societally problematic behaviours (versus neutral or positive behaviours) as being an aspect of illness or disorder rather than identity, it may be prudent to explore how parents view their child’s identity changing when using CE based on the values that are attached to the traits targeted by CE, with and without a known pathology underlying the trait. Our study addresses this by taking data from parents of children with varying cognitive abilities, and exploring how perceptions of CE change based on the traits CE’s are targeting and whether or not a diagnosed pathology is present.

Policy suggestions pertaining to parents’ use of CE interventions have already been put forward [10]. However, our current lack of knowledge makes it difficult and problematic to decide what policies and safeguards ought to be in place, if any [9]. Furthermore data reflecting parent perceptions have been explicitly called for in the CE literature [10] but are still missing. Our study interviewed parents of both cognitively disabled and non-disabled children and we explored how parent’s experiences with varying ability levels of their children affects their views of CE. Most studies that have been conducted thus far have focused on one type of CE, usually the drug methylphenidate [9]. Researchers voiced the need to collect data pertaining to a wide range of CE’s to understand how the type of CE will affect perceptions and opinions [9]. Our study did this by specifically asking if and how the type of CE (natural substances, pharmaceuticals, removable devices or surgical intervention) changes parent’s thoughts towards the use of CE’s. This allowed for direct comparison of different types of CE by the same individuals. Additionally, questioning about various CE’s will allow for in-depth exploration of how enhancement type reflects upon the perceived consequences or benefits of using CE.

A final issue in need of further exploration is that CE is complicated by issues of medicalization [18–20]. According to the definition used in this study, CE is achieved when cognitive abilities are improved above what is considered to be ‘normal-range’ functioning for human beings. Many physicians are opposed to prescribing CE’s on the basis that these products do not treat any illness and are outside the scope of medical practice [13, 15]. Others have reported feeling that they would feel comfortable for those with cognitive disability to use CE products for treatment purposes (therapeutic enhancement), but that it is inappropriate for healthy individuals to be using such products [14]. Where this distinction between ‘cognitively disabled’ and ‘normal functioning’ lies is fully unclear, and will likely be made more ambiguous as our cognitive traits (emotions, intelligence) continue to be pathologized [18, 19]. There is need to explore this distinction, and how CE’s are viewed differently based on ability [9] an aspect which our study explored. This will potentially help to shed light on how views of CE’s differ based on the child’s ability and the labeling of the child’s abilities as pathological or non-pathological.

## Method

### Theoretical Framework

The data collected for this study was analyzed using an ableism framework. Ableism refers to the appraisal of value and worth based on abilities [21]. When using an ableism framework, special attention is paid to implicit or explicit ability expectations and the consequences of having or lacking these expected abilities. Within this study, we thematically analyzed transcripts while keeping an ableism framework in mind. This allowed for a greater understanding of the cognitive abilities parents expect of their child and how these expectations may shape their perception of CE’s. As well, ableism can help to clarify complex constructions of CE’s. For example, a parent may support CE’s because they want their child to be intelligent, but be against it because of the stigma that comes with using medication. The framework of ability expectations and ableism helps to understand and categorize the factors (ability expectations and consequences) that are seen to influence whether or not parents would choose to encourage CE use in their children.

### Study Design

The study design for this project was one of qualitative exploration. A qualitative design was employed because the topic (perceptions of CE) is currently immature. Additionally, the data we were aiming to collect could not be fully captured by quantitative methods—the researchers sought to *identify* influences pertaining to CE perception, which is best achieved through open-ended, nondirective questioning [22]. As to inclusion criteria there were two groups of participants: parents of cognitively disabled children and parents of nondisabled children. Parents of cognitively disabled children were required to have at least one child with a diagnosed cognitive disability that was still considered dependent (i.e., the parent still acted as a caregiver and the child still resided in the parent’s home). We broadly defined cognitive disability as a marked deficiency in cognitive functioning or ability that had been diagnosed by a medical or educational professional. We relied on parents’ self-report of the diagnosis but parents were *not* required to disclose the exact disorder; indeed, some parents chose not to because the clinical diagnosis of their child was rare and publicizing their child’s disorder, even without names attached, could compromise their privacy. Others were comfortable in disclosing their child’s disabilities, which included, for example, autism spectrum disorders, attention deficit hyperactivity disorder, and learning disabilities. Parents of nondisabled children were required to have at least one child between the ages of 3 and 9 years old without diagnosed cognitive disability. This was to create some similarity between the groups despite the discrepancies in age in that all children were dependent on the parent being interviewed for care. As to exclusion criteria any subjects not residing within the cities of Calgary, Red Deer, or Edmonton were not considered. The researchers conducted semi-structured interviews with two groups: parents of cognitively disabled children and parents of nondisabled children.

### Sampling

Participants were recruited via purposive sampling. In this case, purposive sampling allowed the researchers to explore two specific groups in depth (parents of nondisabled 3–9 year old and parents of cognitively disabled individuals). Purposive samples are not representative of larger populations and therefore the findings drawn from this sample cannot be generalized to larger populations however, the data arising from this study may be used as a basis for future research regarding parents and CE’s, and our findings add to the current literature on CE perception [22]. There were two groups of participants: parents of cognitively disabled children and parents of nondisabled children. 6 participants were recruited for each group. In order to meet the inclusion criteria, parents of cognitively disabled children were required to have at least one child with a diagnosed cognitive disability that was still considered dependent (i.e., the parent still acted as a caregiver and/or the child still resided in the parent’s home). We broadly defined cognitive disability as a marked deficiency in cognitive functioning or ability that had been diagnosed by a medical or educational professional. We relied on parents’ self-report of the diagnosis, but parents were *not* required to disclose the exact disorder; indeed, some parents chose not to because the clinical diagnosis of their child was rare and publicizing their child’s disorder, even without names attached, could compromise their privacy. Others were comfortable in disclosing their child’s disabilities, which included, for example, autism spectrum disorders, attention deficit hyperactivity disorder, and learning disabilities. Parents of nondisabled children were required to have at least one child between the ages of 3 and 9 years old.

Ideally, the researchers aimed for the children of both sets of parents to be of the same age to maximize comparability between groups. However, due to time and resource constraints, we were unable to locate a sufficient number of parents of children with cognitive disabilities between the ages of three and nine. Therefore, we instead required that the cognitively disabled children still be dependent; that is, living with the parent and receiving some level of care-giving from the parent. This was to create some similarity between the groups despite the discrepancies in age in that all children were dependent on the parent being interviewed for care.

Individuals who lived outside of Calgary, Edmonton or Red Deer, who had a spouse participating in the study, and/or those who failed to meet the inclusion criteria were excluded from being eligible to participate in the study. Participants were identified via personal and professional contacts of the researchers. Initial contact was made via email or telephone and interviews were conducted face-to-face.

### Data Collection

#### Measurements/Instruments

This study was conducted using semi-structured interviews developed by the researchers. A 6-question, 4 sub-question research protocol was developed by incorporating themes pertaining to CE perception that were identified during a review of the current CE literature and employed. Questions were open-ended to encourage in-depth exploration of parent’s perceptions of CE with respect to their own child’s identity, the type of CE used, and external pressures to encourage or discourage CE use. The protocol served as a template for the interview; additional, unscripted probing questions were asked as the interview progressed to clarify or further explore participant’s responses.

#### Procedures

Data were collected using face-to-face semi-structured interviews lasting approximately 30 min. A semi-structured procedure was advantageous because it allowed the researcher to control the line of questioning and probe further as necessary, and because participants are free to incorporate personal or hypothetical examples and narratives if they feel it contributes to their answering of the question [22]. Additionally, this method allowed for impromptu clarification questions, which serves to strengthen qualitative validity.

### Data Analysis

The data collected for this study was analyzed using an ableism framework. Raw data from interviews were analyzed using content analysis as described by Elo & Kyngäs [23]. We used ATLAS.ti^©^, a qualitative data analysis software (CAQDAS) [24, 25], for generating the qualitative data. Interviews were transcribed, read, broken down into themes, organized into larger categories, and then the themes and patterns were interpreted as a whole [23]. Both authors engaged in analysis, and codes were cross-checked between the two researchers to get a sense of inter-coder reliability [22]. Our analysis was both deductive and inductive as well as iterative—the investigators sought out the presence of certain themes based on review of the literature; however, themes were also generated as they were seen to emerge from the data. The interviews were re-analyzed twice after the initial coding to ensure that themes had not been overlooked. To increase reliability of the analysis, the co-investigator provided a definition for each code or theme to ensure that the meaning of a code did not shift as the analysis progresses [22]. Once themes were gathered, they were organized by how they relate with one another and how the themes generalized into larger patterns. Finally, the overall interpretations of these themes and patterns were recorded. Throughout the analysis, the investigators engaged in peer-debriefing and personal reflection to strengthen the validity of the findings [22].

#### Data Presentation

All names used have been changed. ‘ND’ denotes the participant having a nondisabled child, whereas CD signifies that the participant’s child is cognitively disabled. Where significant differences were noted between parent groups for a given theme, there will be a subsection included called ‘Comparison of Parent Groups.’

### Limitations

Given the nature of this study, social desirability bias is a definite possibility. Interviews were conducted face-to-face, and though participants were guaranteed anonymity if their views were disseminated, they were not anonymous to the researcher. As was illustrated through the quotes provided by parents (see Results), the idea of forcing a child to do something that may harm them or acting in a way that shows they do not accept their child was seen as highly negative. Therefore, parents may have felt stifled in sharing their true opinions for fear of being stigmatized. However, this method of data collection was chosen because it allowed for exploration and identification of relevant issues—something that was identified as being a gap in the CE literature [1, 9, 12]. The face-to-face, interactive format allowed participants to ask clarification questions, which was useful given that it was a novel topic to several of them; additionally, it allowed the researcher to ask probing questions based on the responses of parents.

The ages of the participant’s children were not the same for both groups due to the constraints of the study. Had the groups been within the same age range, comparisons could have been made between groups without considering age difference as a factor in forming their opinions about CE’s. However, due to the time constraints of this study, we were unable to find enough participants with cognitively disabled children whose child fell between the ages of three and nine. Nevertheless, all participants’ perspectives were informative regardless of their child’s age, and parents with older children still chose to share their general thoughts towards parents encouraging or forcing their child to use CE’s.

Despite its limitations, this study provided greater insight into the factors that shaped CE perception. Using this insight, further research can be conducted in a format that reduces social desirability bias (i.e., an online questionnaire or mail-in survey) to gauge how prevalent these practices and beliefs are.

### Ethical Considerations

This study was approved by the Conjoint Health Research Ethics Board (CHREB) at the University of Calgary on August 17, 2012. Individuals were required to read and sign the informed consent form mandated by CHREB before participating. Participants were informed of their role in the study, what was expected of them, expected harms and benefits, and that their willingness to participate is completely voluntary and may be withdrawn at any time for any reason.

### Funding

This project was completed in order to fulfill the requirements of an undergraduate honour’s thesis and therefore received no funding.

## Results

A total of 12 parents were recruited and interviewed for this study. Each participant came from a different family unit. Six were parents of children with varying cognitive disability. As discussed in the method section of this paper, parents were not required to disclose the exact disorder or disability and some chose not to in order to preserve their own privacy as well as that of their child. However, broadly speaking, cognitive disabilities ranged from learning disabilities to pervasive and severe limitations that required full-time care giving. For those comfortable in disclosing their child’s condition, disabilities included, for example, autism spectrum disorders, attention deficit hyperactivity disorder (ADHD), and learning disabilities affecting reading and writing. The remaining six participants were parents of children between the ages of three and nine that had no diagnosed cognitive disability. All but one of the participants was female and all resided within Alberta, Canada. A table summarizing the characteristics of participants and their children is provided below (Table 1).

**Table 1:** Characteristics of interview sample

	Gender	Child with cognitive disability?	Ages of children^a^
01	Female	Yes	4
02	Female	No	4
03	Female	No	3
04	Male	Yes	22, 24
05	Female	Yes	25
06	Female	No	3
07	Female	Yes	8
08	Female	No	9
09	Female	Yes	8
10	Female	No	4,6
11	Female	Yes	10
12	Female	No	3

^a^Ages of children that fit within the study criteria

Theoretical saturation was found to be reached in the groups of parents with nondisabled children; however, likely due to the varied experiences that parents of cognitively disabled children faced, saturation was not achieved with this group. These parents brought varied perspectives based on the unique nature of their child’s disabilities and the regimens that they implemented in order to mitigate the negative effects of the disability. Many of them had real-life experience using products that may be used for cognitive enhancement purposes in nondisabled individuals, such as removable devices for manipulating oxygen intake to promote better cognition or pharmaceuticals to improve focus, anxiety and aggression. Therefore, perspectives from parents with cognitively disabled children were grounded in personal experiences and tended to be more nuanced than those of the parents with nondisabled children. For this reason, we would have ideally liked to interview more parents of cognitively disabled children in order to capture their diverse perspectives and experiences.

Analysis of the interviews revealed six primary overarching themes relating to conceptions of CE: meanings of health and treatment; the role of medicine; harm; the ‘good’ parent; normality and self-perception; and ability. Aspects of these themes were often in direct contradiction of one another, illustrating the complexity of the topic.

### Meanings of Health and Treatment

Parent’s conceptions of what health means and the more specific subdivisions of it—what is disability or disease, what is treatment, how does enhancement relate to health—were influential factors in deciding whether or not CE use was acceptable. Judgments of health and disease/disability impacted parent’s thoughts regarding who should be allowed to use CE’s, who is allowed to decide if CE’s can be used, and if CE’s were considered ‘enhancement’ (as defined at the outset of this paper) or ‘treatment.’ Parents unanimously agreed that products that could be used for cognitive enhancement could be acceptable when disability was definitely present so long as these products were safe and provided a clear benefit to the child. Parent’s initial reactions to CE as a means to bring their child *above* an average level were near-constant reiterations of the same idea, namely that they could not comprehend why a parent would give their child CE products without disability or disease:

“Um, surgical [cognitive enhancement], I’m thinking, cause I feel like [my daughter] is average for cognitive, or could have some strengths, so then I think

why would I do surgery if she’s just going to be an average child? Why would I put her through surgery? So, to me that doesn’t really make sense. Like it’s kind of like taking her life in my own hands and making decisions that really are not appropriate when she is functioning at an adequate, capable level.” (Natasha, ND)

“And I just, I just don’t think—I mean, I don’t even want my son to be taking them, so I certainly wouldn’t—no, I wouldn’t say yes to the stimulants for a typical person.” (Kyla, CD)

Parents recognized that there were serious risks and drawbacks to using CE products in any child, but these risks were considerably more discouraging if their child was not experiencing some form of cognitive disability or deficiency. This was particularly true of parents who did not have a child with cognitive disability.

Where CE was conceptualized as a *treatment* rather than some sort of cognitive booster, parents were more open to using the products that had been outlined to them. Despite being provided a definition of CE as any product which brings the user *above* a normal or average level, both groups of parents tended to discuss and repeatedly return to the use of CE products in order to bring a child *up to* an average level. Again, the label or diagnosis of a cognitive disability was important to parents; the actual medical definition of what constituted disability held significance as well. Diagnostic criteria seemed to give parents a sense of ease towards using CE products because it helped to ensure that the consumption of these products was not based on issues of self-esteem, distorted expectations, or competitiveness. Through probing, however, some parents did reveal that the presence of a medically-defined disability may *not* necessarily be required to provide clear justification for CE products. What was more important was that these products be used in a treatment-like sense. That is, even if there was no clinically diagnosable syndrome or medical issue, parents could be motivated to use enhancement if their child was experiencing struggle, even if they were unable to obtain a diagnosis for disability.

The type of CE product influenced if use was acceptable. Parents implicitly categorized CE products based on whether or not they were medical treatment. Treatment products were those that parents viewed as being intended for disease or disability management. Products that did not fit this categorization were seen as more of a lifestyle choice that enhanced or promoted overall well-being. Products that were viewed as being medical treatment (most often pharmaceuticals and surgery) were viewed as less acceptable to use for CE compared to products that were not (natural products and possibly removable devices):

“If it was like prescription, something prescribed, I don’t think I would do it. If it was more of a natural supplement […] like multi-vitamin, omega 3, that kind of thing, I might be more willing to consider […] I just don’t like giving them any kind of medication that they don’t necessarily need.” (Hannah, CD)

It is important to note, however, that parents did not necessarily condone these products for improving cognitive abilities above a normal level; some simply expressed that they felt non disease-relievers were the better choice when comparing CE products. For the products grouped as treatment, parents felt that the distribution of these instruments for CE ought to be regulated and monitored by health professionals because these should be used for disease or disability relief, a point that will be expanded upon further on in this paper. As the quotes above illustrate, this is largely tied to the perception that these products carry a significant risk of harm, which will be discussed in-depth in the next section. Non treatments did not carry the same perception of harm, though two of the participants voiced their skepticism that natural products were always less risky than pharmaceuticals. Parents expressed being more attracted to natural products, and generally voiced less concern about regulating the use of the non-disease relievers.

For parents who were open to any form of CE—whether the product was disease- relieving or not—conceptions of health remained influential. Those parents (three of the twelve participants) viewed CE use as a tool that could promote a sense overall well-being for the user, provided that the enhancements were safe and effective. One parent in particular argued that if the child chose to use CE’s (given that they were competent to make that decision), that process could end up improving that individual’s physical and mental health through boosting their self- confidence, independence, and opportunities for success. Those more open to CE use viewed having a sense of overall well-being as an important part of health, and vice versa. Therefore, CE above an average level could be considered as a tool to improve health if used for the right reasons, even if no disability or disease were present.

### Harm

Harm was a primary concern for parents when forming their opinions about CE. In this case, harm refers to negative physical consequences arising from using a CE product. Every parent discussed harm as being a deterrent to using CE’s independent of the interviewer prompting. Each parent acknowledged that CE carried a significant risk for harming their child, and this was generally their first consideration when deciding whether or not they would allow their child to use CE products.

Parents feared the possible side effects of CE products, particularly pharmaceuticals. Participants with cognitively disabled children often had first-hand experience with a number of these pharmaceuticals:

“We’ve given him other drugs that make him so sleepy he can barely lift his head off the—I just, I don’t like giving him the drugs, I don’t like changing his body, I don’t like changing the way he is. I think we are who we are. Unfortunately, I feel a little bit trapped, right, in that respect […] I would like to take him off all of that, altogether, if I could.” (Kyla, CD)

Participants of nondisabled parents based their knowledge on observing children who did have cognitive disabilities, often a child that had ADHD and was using Ritalin. More generally, parents were hesitant of any CE because “everything has side effects [… it would] depend on what the side effects [were].” (Jillian, ND) In the absence of a medical reason to use potentially damaging products, parents did not see the potential benefits as outweighing the potential harms. As shown by the above quotes, the presence of a cognitive disability was not necessarily enough to counteract the harm that accompanied using CE products either.

The lack of evidence or personal knowledge about the harms of CE products also acted as deterrents. Parents wanted the products to be “double-blind studied” (Victoria, CD) and they expressed the desire to be informed of “what the research had been” (Hannah, CD) regarding the product in question. Five parents explicitly articulated that they worried about harm appearing later on that we are not currently aware of. Parents identified this general distrust of the safety of these CE products as a major reason to discourage their children from using them. Additionally, a few of the parents expressed that invasiveness held greater potential for harm, with one parent articulating that “the more invasive [enhancements] are, the less palatable they are.” (Roxanne, CD)

Parents expressed preferring certain types of CE products and alternatives because they were viewed as being ‘no-harm’ approaches. The idea of making lifestyle changes or using natural products (to some extent) was identified by a few as being more appealing because they viewed it as having either a beneficial effect or no effect at all. Ultimately, harm was a primary consideration for parents. In the absence of cognitive disability, parents were unwilling to even consider CE’s if they had not been guaranteed that there would be no physical harm to their child from using those products.

#### Comparison Between Parent Groups

Parents of cognitively disabled children and parents of nondisabled children both felt that harm was a primary concern. However, parents of cognitively disabled children were able to offer their thoughts on certain CE products (mostly pharmaceuticals) from long-term personal observation of the harms that can arise from using these products. Parents of nondisabled children often had to rely on anecdotes based on other parent’s children and often admitted that they had limited knowledge of the side effects of various CE products because they had never needed to consider them for their own child. Despite these differences, however, their overall opinions concerning harm were the same: potential harm to their child was unacceptable in the absence of medical need, and that certain CE interventions (pharmaceuticals and surgery) carried a high risk of harm.

### The Role of Medicine

Parents were uncomfortable making decisions regarding CE for their children without aid from the medical community. This included members from medical research, health education and health practitioners. Health practitioners, particularly physicians, were the ones most commonly identified as being appropriate for making decisions regarding CE. Parents supported this by arguing that physicians have greater knowledge of the products as well as the harms and effectiveness of CE’s in addition to having an objective view and diagnostic tools (such as diagnostic criteria set out by the DSM-IV) to determine if CE is appropriate:

“I think if you’re going to be using surgical intervention, or pharmaceuticals, there should be—whether it’s DSM-IV criteria, or whatever, there should be guidelines around who can assess and what the criteria are for diagnosis, that somebody is actually going to require these interventions. Alternative therapies are harder.” (Kathleen, ND)

Participants expressed concern that without the aid of a physician, individuals may inaccurately diagnose themselves with a disorder and use CE products without fully appreciating the associated risks. A number of parents with cognitively disabled children warned that “self-report [of cognitive struggles] is not a very accurate way of describing” (Roxanne, CD) and that those with low self-esteem often had distorted views of what would actually constitute a disability.

While parents wanted the involvement of health professionals in making CE decisions and agreed that diagnostic tools were useful, they were hesitant about implementing inflexible guidelines to rely on regarding access to CE products. Participants stressed that situations concerning abilities and treatment were often complex and/or unique. In order to address this, it was suggested that health care practitioners assess each case individually and respond based on the specific situation that the child was facing.

Parents also raised concerns that broad-sweeping decisions based solely on ability levels and diagnostic criteria could leave children who failed to meet diagnostic criteria without help and force treatment upon children who *did* qualify for diagnosis, even if they did not want it.

“I don’t think that it’s necessarily right for the general population to decide for everyone else what’s right, I mean, that goes back, I mean you just go back to Nazis, and that sort of stuff […] it could be argued that that line of thinking, of making a […] super race, that […] it kind of tends off in that direction.” (Robert, CD)

Though most parents were generally uncomfortable with the idea of CE purely to bring cognition to an above-average level, several acknowledged that there may be situations where it was still in the best interest of the child; therefore, health professionals should be flexible and willing to assess each individual situation.

Parents communicated that it would be difficult for them to come to a conclusion about CE’s without extensive research regarding the safety and effectiveness of various products. Parents used their own knowledge to inform their opinions, but for those who had limited experience with CE products (or had never heard of them), they wanted to look at the research that had been conducted before even *considering* using any CE’s on their child. For parents of children with cognitive disabilities, some worried that the public was not educated enough with respect to the safety, effectiveness and proper use of some of the products used for CE:

“I mean, I think we all do it, I think we go ‘oh, okay, well, you know I think I would’ve done a lot better in school if I had had these drugs, I would have been able to focus better,’ but that’s not the case. When you have a kid who’s as bad as [my son] is, that helps you to put a lot of stuff in perspective […] Taking Ritalin and Biphentin, well, they’re amphetamines. […] I wouldn’t say yes to the stimulants for a typical person.” (Kyla, CD)

Knowledge held an important role in conceptualizing CE’s and their use in children and parents wanted this knowledge available through their physicians, education, or public forums.

#### Comparison of Parent Groups

Both groups of parents felt more at ease with a physician supervising the use of CE interventions and expressed a desire for rigorous research pertaining to CE’s to inform their opinions.

However, a few of the parents with more severely cognitively disabled children (autism spectrum disorders and severe ADHD) additionally argued that individuals are prone to inaccurately self- diagnose disability when undergoing struggle, particularly if they lacked close experience with disabled individuals. They related this to their own encounters with struggle and their child’s disability. These parents felt that others often did not understand the difference between everyday challenges—feeling tired and unable to focus—and the pervasive functional impairments that their own children faced due to their cognitive disability.

### The Role of the ‘Good’ Parent

Throughout the interviews, parents related their responses about CE use to how they as a parent ought to be responding and acting. It became obvious that feeling like a ‘good parent’ was neither straightforward nor easily achieved when deciding to use CE’s. The ‘good parent’ was seen as one that accepts their child, supports a well-rounded development and sense of well-being, protects their child from harm and stigma, and acts in accordance to their own beliefs rather than acting because of outside pressures or forces. Conversely, a ‘bad parent’, according to the participants, pressures their child, exerts excessive control, neglects the possibility of harm and disregards the child’s best interests or well-being.

CE’s were often seen as being at odds with giving children a sense of acceptance. Parents worried that if they were to encourage or force their child to use CE’s, they would run the risk of their child feeling unloved, unworthy, and/or pressured to do more than they feel comfortable with.

“I think that as a mum I would encourage my children to succeed at whatever level they are at, versus […] trying to force them into being somebody they don’t want to be […] that’s something that they learn from their parents, that they are not allowed to be who they are, that they have to be someone that their parents agree with, or want them to be, and I think that […] it might cause kids to not like who they are, or think that they have to be somebody better or more or less of some characteristic in order to have love or friendships or success, and […] I don’t think that’s healthy, I don’t think that’s the way that people should grow up feeling.” (Alexia, ND)

“I would hope you would work on self-esteem and confidence and accepting themselves as they are, and playing up their strengths […] By doing things to enhance cognitive abilities, you’re just kind of highlighting that they aren’t [good enough].” (Kathleen, ND)

Parents also expressed that the lack of acceptance shown by pushing CE’s on their child had other negative implications, like failing to develop the child’s natural interests and talents, causing the child to miss opportunities, or to obscure the child’s view of who they are.

“I think it would be almost just trying to make your child how you want them, like rather than just accepting them as how they are, whether they’re […] advanced cognitively or delayed, or just, you know, like average […] I feel like you’d be trying to change their identity, almost? […] I don’t understand why people would wanna do that. […] Just let your kid be a kid. […] Let them think how they’re gonna think and be how they are.” (Jillian, ND)

The parents of both disabled and nondisabled children felt that as parents, it was their job to ensure that their child felt accepted for who they were.

Parents viewed themselves as protectors against physical and emotional harm for their children. This was especially salient for the participants with younger children. Because CE’s opened up the possibility for harm—or worse, harm without a perceived benefit—every participant was averse without assurance that the CE would be safe for their child to use, as was discussed in a previous section. Additionally, parents acknowledged that there is stigma associated with many CE products, and that this should be avoided if possible. The stigma attached to products that could be used for CE was a deterrent for encouraging their own child to use them, especially when the enhancement would be highly visible to the public.

Parents supported a balanced, well-rounded approach to their child’s development. However, for some parents, this worked in favour of CE use, whereas others viewed CE’s as creating imbalance or an incomplete development of their child’s overall identity. One participant felt that using CE’s should be a personal choice and may help individuals achieve greater balance (though he also acknowledged possible dangers of CE elsewhere in the interview):

“But I think that if the technology is there for them to do it, then it should certainly be made available to them if they want it […] I would say that […] from the perspective that I think we’re talking about here, that it is best that the goal be to make that person, for lack of a better term, to make that person whole.” (Robert, CD)

Other parents argued that focusing too much on cognitive abilities would detract from other areas in the child’s life:

“Being very skilled at math makes you skilled at math, but it doesn’t necessarily make you a better person. […] It doesn’t make you more compassionate, it doesn’t […] make you more kind, more sociable. […] Being good at math, or TOO good at math […] isolates you from the group.” (Roxanne, CD)

Parents expressed that it was their responsibility not to impede their child from figuring out and achieving their individual identity. Several reported feeling that in giving their child CE’s, they were pushing or coercing their child to be a certain way that may not reflect what the child wants or feels is right for them.

The parents interviewed expressed that one of the goals of parenting should be to do what is best for their child without exerting excessive control over them. Therefore, parents felt that *forcing* their child to take CE’s would be unfair, particularly if it is for a reason that was self- serving to the parent:

“I think the biggest deterrent is what we just talked about, about the competition. About there being reward for excelling in certain areas and the kind of culture and environment that creates. And the competition that could ensue between, especially with parents over their children, when their children are not able to make those choices themselves, but the parents are making the choices. I think that’s dangerous […]” (Roxanne, CD)

Pushing CE use on a child was seen by many of the parents as pressuring their child to be someone and this was viewed as being a very negative force for the child. However, as children aged, parents wanted to allow their children greater decision-making power that was reflective of their growing competency and independence. Some discussed how it was important that they respect their child’s desires and ideas, so if their child truly understood CE’s (the risks, benefits, alternatives) and wanted to use them, they would be willing to discuss the possibility with them:

“I think as they get older, it becomes a little bit more of what they want, and not as much as what *I’m* saying, as a parent. And if it’s truly driven by their need and they’re informed and understand […] I could support their decisions, but I don’t think it would be my decision to make for them.” (Kathleen, ND)

“[…] Everybody’s opinion in my family is valid, so if somebody says I would like to put this on my head so it makes me think clearly, we would look into that to make sure that it is a positive outcome.” (Marie, CD)

What was not clear was the age at which parents thought the average individual *would* be capable of making that decision and understanding the ramifications of it. However, parents of nondisabled children (whose children were between the ages of 3 and 9) were quite clear that they felt using CE’s during this time in their child’s life *would not* be the decision of the child, but the decision of the parent.

Parents were concerned with providing their child a high quality of life and overall sense of well-being. Things related to CE’s that could threaten well-being have already been discussed—harm, pressure/coercion, failing to allow the child to be their true self, and creating feelings of rejection and worthlessness. However, there were some ways that parents could imagine CE’s having a positive effect on well-being. For example, if their child was struggling in a particular area and using CE’s lessened that struggle, it would likely increase their child’s confidence and decrease their frustration. Because their children had experienced significant struggle, parents of cognitively disabled children appeared more concerned about the damaging impacts of being unable to do something—losing out on opportunities, low self-esteem and self- worth, isolation, and poor quality of life:

“[…] If it’s a cognitive thing, it could improve someone’s life […] from a career orientation, if somebody may have a lot more options available to them […] I personally know of a young man who’s a super nice guy, […] but he has a significant cognitive issues that cause a lot of obstacles for him to be gainfully employed in the workforce. So all those things that go with that—lack of revenue that allow you to […] buy things and do all that stuff […] that we all become accustomed to—that you can’t do. So that would be a significant one. Another would even be socially. […] he would love to have a girlfriend. But that’s really difficult for him.” (Robert, CD)

“There’s a lot of suffering that happens when you’re different. There’s a lot of suffering that happens when you’re not able to do things as well as most everybody else is. And there’s a real lot of suffering that happens […] when you’re not physically able to be identified as having deficits, because an expectation exists then that you are normal.” (Roxanne, CD)

Parents of nondisabled children viewed struggle as more of a part of growing up and as a necessary life lesson, though they did acknowledge that they would view the situation differently if their child had a disability and that their own child had not experienced struggle to an extent that it impacted their quality of life.

Parents from both group often preferred to take an alternate approach to decreasing struggle rather than using CE products, like spending more time with their child or hiring a tutor. They viewed these approaches as less invasive and as promoting better self-esteem for their child. However, a couple of the parents of cognitively disabled children expressed frustration that these alternate approaches were not feasible for their own child because of failures by the educational or health system.

Participants generally felt that parenting should not be dictated by external forces such as pressure from other parents or societal norms. When asked how they would react if a significant number of other parents were using CE’s on their children, these were some of the responses:

“I guess I would think about it, but […] I’m really confident in the parent I am. […] Since [my son’s] been born, I’ve realized that […] I needed to find my own empowerment as a mum, stick to that, and stick to my intuition and I think […] would think of it, but I would think, ‘is it something that I should consider?’ But I would not be swayed by it, I think that [my husband] and I would sit down […] and make a decision based on what we believed, and what we thought our kids needed, and what was best for them.” (Alexia, ND)

“And I’m not one to follow the trends, so it wouldn’t be just because so and so’s kid is taking […] this particular […] herbal medicine to make him stay focused, or read better that doesn’t mean I’m gonna go try the new trend on my child. I may look it up and see what the whole trend is all about, but […] I think each to their own, and I think everyone’s entitled to bring their children up the way they think it’s right. As long as there’s no harm! (*laughs*)” (Marie, CD)

“I don’t allow myself to be affected a lot by that kind of a peer pressure when it comes to my child. I dunno, maybe it’s my age (*laughs.*)” (Megan, ND)

Parents expressed feeling quite confident in their own beliefs towards CE, and that the most likely impact that CE use in other children would have would be that they would want to seek out more information about CE’s. Others did acknowledge, however, that other’s parenting practices (forcing their child to use CE’s) would be a concern if it began to disadvantage their own child:

“Really of no consequence to me […] unless [CE use in others] affects myself or my son directly, I don’t care. And if it did affect him directly, then it would depend on how that would impact him. If I thought that it impacted him in any sort of negative way, then I’d wanna take a look at it.” (Robert, CD)

“I would definitely look at […] what difference it was making for my kids, if they were at the top of the class and now that everyone’s taking them they’re at the bottom of the class, I would definitely talk to the teacher more to just try and get the situation as to what’s happening with that. But I think it would take a pretty significant amount of change for me to do anything about that.” (Bella, ND)

Parents did not necessarily see disadvantage to their child as *necessitating* CE use, but it was cause for greater investigation of CE products with some participants.

Many parents saw CE use as a threat to their ability to parent the way that they wanted to. Most of them explicitly expressed that they did not want to encourage their child to use CE products because they felt that it would make their child feel as though they were not acceptable ‘as is.’ However, parents were also concerned with providing their child with a good quality of life, and they recognized that if their child was put at a disadvantage by *not* taking CE’s, quality of life was threatened because their child’s opportunities were limited. Most of the parents did not wish to perpetuate competitiveness for cognitive performance, but they acknowledged that competitiveness was a reality in current society and could pose an issue. From the interviews, it was clear that CE’s put many participants in an uncomfortable position because CE’s could force them to choose between two values that were important to them as a parent.

#### Comparison of Parent Groups

As was the case with other themes arising in this study, differences between participants with cognitively disabled children and participants with nondisabled children tended to be grounded in personal experience. Many parents of cognitively disabled children put more weight on the damage that struggle can have on a child because their own child had suffered significantly due to their inability to perform certain tasks or act in certain ways. Parents of nondisabled children did not view struggle as being quite so harmful and indeed, many conceded that this was likely *because* their child had not experienced significant struggle. Both sets of parents generally preferred lifestyle changes compared to more invasive interventions (i.e., having their child ingest something or undergo a medical procedure) to improve cognitive abilities where possible. However, parents of nondisabled children did not mention concerns that these alternatives might be unavailable and therefore did not consider how they may react if potential CE products were the only option. Alternatively, some of the parents of cognitively disabled children had felt forced into using pharmaceuticals to improve cognitive functioning because the setup of our health and educational system simply did not provide any support for using alternative approaches.

### Normality and Self-Perception

The normality theme refers to parent’s discussions of the consequences of their child being considered (or viewed by most) as ‘normal.’ Mentions of normality almost invariably related to how a child perceives themselves and what that meant for the child’s self-worth and feeling of belonging. Self-acceptance and ‘fitting in’ were crucial considerations in conceptualizing enhancements and CE’s were discussed as having potential to both promote and detract from these goals. Parents could foresee changes in normality and self-perception brought upon by CE’s being an encouragement *or* a discouragement to using CE products.

Parents were quick to point out that being seen as abnormal because of a deficiency was stigmatizing and isolating. Isolation and failing to ‘fit in’ was seen as causing great suffering; therefore, achieving an appearance of normality was something that could be considered as a reason to use CE products. However, even when isolation and struggle were present, parents were hesitant to use any CE enhancing products in the absence of disability or disease. This sentiment was brought up from personal experience of parents with cognitively disabled children and echoed through the speculation of parents with nondisabled children. Having their child feel like they fit in with their peers was important to parents, with some expressing that it gives the child a more positive view of themselves and a more balanced development.

“I think he just wanted to believe that you *can* cope. Just like *everybody* else, right? You want to feel quote ‘normal.’ You want to be able to […] be like your friends, I guess.” (Hannah, CD)

Parents argued the other side of normality as well—if abilities were too far *above* normal, it could have damaging impacts on social- and self-acceptance. This quote was from a mother who had been considered an academic prodigy in her childhood and now had a daughter with cognitive deficits:

“From my own experience, enhancements, cognitive enhancements, get you noticed. And it becomes how you are identified by others and therefore it becomes how you identify yourself. And you use those enhancements to […] seek fulfillment, to get attention, to […] feed your self-esteem. All of those things […] And you can become quite one-dimensional. And so, you’re just the brain […] you don’t participate in sports, and you’re not social, and you’re not a good friend and all of those things.” (Roxanne, CD)

She and a few other parents argued that in trying to achieve a life for their child that was balanced emotionally, socially, mentally and physically, straying too far from normality (whether above or below) was a major barrier.

Participants expressed their struggle in helping their child to feel like ‘part of the group’ without causing their child to perceive themselves as deficient, or needing to be fixed. Some of the parents were attracted to CE products in order to help their child feel more normal, because the real or perceived abnormality was harming their child emotionally and socially. However, parents found it hard to strike a balance between achieving normality while still encouraging their child to accept themselves for who they were. Both groups of participants expressed worry that encouraging their child to take CE products would leave the child feeling ‘broken’ or incomplete as a person, even when the intention of the products was to mitigate their child’s suffering.

Several parents brought up that constantly striving for an above-normal performance fostered harmful levels of competitiveness. They expressed that through demanding top performances from their child, they felt their child would begin to view themselves only in terms of achievements and they would not be able to explore other aspects of life.

“I just would think that they’re pressuring their kids to […] not be kids. […] I don’t agree with putting your kid in every extracurricular activity, making them the best of the best, they need to be an olympian, they need to be the smartest kid in the class, they need to be a doctor… […] why can’t they just choose what they wanna do, why can’t they be kids for a while? […] I think it puts too much pressure on them to try and be the very best, not just the best that they can do. […] I don’t think it’s fair to put them against each other.” (Bella, ND)

With a focus on competition and performance, many parents worried that children would be prevented from finding and exploring who and where “they are *meant* to be.” (Kathleen, ND)

### Ability

Finally, CE centers on enhancing ability; unsurprisingly, ability expectations and ability consequences were salient themes in this study. Participants identified a number of abilities that were important for their child to possess as well as the consequences that would follow for lacking that ability. Parents also discussed the implications of having ability expectations for children—particularly any negative impacts that this could have on their child.

When cognitive abilities were severely compromised—enough to warrant a diagnosis of being disabled—parents generally found CE products acceptable. As has been discussed throughout this results section, parents expressed that this was because of the negative consequences arising from below-average ability: low self-confidence, limited opportunities and social isolation. Other negative consequences for the child that parents identified included being reprimanded by teachers for being unable to focus; being frustrated because they cannot complete a basic task; and being denied opportunities because they are unable to perform well in school.

Interestingly, parents tended not to focus on the cognitive abilities that CE’s would target; rather participants were most interested in the consequences of having or lacking such abilities. This went in favour and against CE’s, depending on the consequence. For example, CE’s were less acceptable when the heightened ability would make their child feel that their parents did not accept them, and CE’s were more acceptable when the improved ability would allow the child to integrate into their peer group successfully. The cognitive abilities themselves were not of primary importance to parents.

Parents did have some ability preferences for their children, however. These were generally expressed by parents of children with cognitive disabilities. They hoped that their child would be able to communicate, learn, gain independence (to the extent that was possible given their disability), and to be able to ‘cope’—that is, to function on a day-to-day basis without suffering. Parents discussed these abilities as being major contributors to a good quality of life for their child, and these were the abilities that were seen as the most essential to parents. Aside from these ability expectations, participants generally expressed that parents should not be expecting any more than their child’s natural ability.

Most of the participants in this study expressed that the most important abilities were the ‘natural’ abilities of their child. The term ‘natural’ was used repeatedly by parents throughout the interviews, and may suggest only those abilities that their child was born with; however, given that abilities are shaped by a number of forces that children are exposed to—education, life experiences, family dynamics—it seems more accurate that parents viewed ‘natural ability’ as abilities that developed out of the child’s own interest or without what they perceive as excessive intervention. Parents viewed these abilities as something that should be cherished:

“I would want my kids I guess to pursue whatever they’re naturally inclined to, so, if they have strong academic performance, that’s great, that’s where they’re meant to be, if they don’t, maybe they’re meant to be […] pursuing the arts, or trades or whatever that might be, so I think we are made the way we’re supposed to be made.” (Kathleen, ND)

Participants from both groups felt that expecting only their child’s ‘natural’ ability signified that they accepted their child for who they truly were. Minor cognitive struggles were seen as a part of natural ability and were still valued by parents as being a part of their child’s identities. However, aspects of cognitive *disability* which parents felt lowered their child’s quality of life did not seem to be considered as part of their child’s identity, even though these abilities would be considered natural as well.

Parents tended to view ability expectations as having negative impacts on their child. A number of participants expressed that putting pressure on children to have certain abilities threatened children’s feelings of self-worth and acceptance (see ‘The Role of the ‘Good Parent”, above). Some expectations that were identified include increased “compliance and [focus]” (Hannah, CD); thinking in a prescribed fashion; and being expected to perform above an average level on tests, assignments, and reading comprehension. These were not only expectations for the child; parents were expected to be able to develop these abilities in their children. Parents identified the sources of these expectations to be from other parents, teachers, and more broadly, the structure of society:

“If you can call it peer pressure, which it is, but […] at an adult age, there’s a lot

of […] ‘what does society expect from you as a parent?’” (Victoria, CD)

Parents of cognitively disabled children brought up these expectations more often, usually referring to personal experiences where their own child had suffered because they failed to meet ability expectations. Parents that discussed pressures to achieve above-average ability argued that these expectations both stemmed from and contributed to competitiveness in society—competing for opportunities and competing to be viewed as more accomplished than others.

#### Comparison of Parent Groups

Both sets of parents viewed ability expectations as harmful and tried to limit their own for their child by saying they expected only ‘natural’ abilities. However, parents of nondisabled may have been taking some of the more ‘fundamental’ abilities as a given, as only parents with cognitively disabled children mentioned that it was important that their child be able to communicate, learn and cope with everyday life. Parents viewed abilities that did not detract from quality of life as valuable and essential to their child’s identity, whereas abilities that did detract from quality of life were not seen as part of their child’s identity and could even be considered as a barrier to their child’s identity.

## Discussion

This study revealed a variety of complex attitudes that parents of nondisabled and cognitively disabled children held towards CE use for their children. Parents were generally hesitant toward their children using CE products. They related this hesitancy to issues of disability, harm, confidence, competitiveness, and acceptance from peers, parents, and self. Participants tended to express feeling that given their current situation and the state of CE products, they would not feel the need to encourage their child to use CE’s. However, situations were identified that could promote CE use—if their child was struggling significantly with cognitive tasks, if their child felt alienated due to differences that could be mitigated by CE, if their child expressed they wanted to use CE’s (after a certain age), or if CE’s are proven to be completely safe as well as effective. The findings of this study complement and enhance preexisting CE literature.

Physical harm resulting from CE use was identified as a central concern by participants, which echoes the findings of Banjo et al.’s (2010) study using physicians [13] and Frank et al.’s (2012) study featuring university students [14]. This study adds to the discourse of harm with CE use by exploring a variety of CE types (natural products, pharmaceuticals, removable devices and surgery) rather than only one specific product, as has been common in previous studies exploring CE perception [9]. Additionally, this study sheds light on how participants felt harm related to their role as a parent. Parents viewed themselves as being responsible for avoiding harm in their child’s life wherever possible and risking harm for their child for the purposes of CE was seen as unacceptable to all participants in this study.

This study enriches existing data pertaining to identity and CE. Like participants from previous CE studies, the parents interviewed here expressed that CE could impact a person’s core identity. However, parents were less concerned by the actual CE product eliciting an inauthentic self, as has been identified in other CE studies involving university students, their parents, and health care professionals [7]. These parents were concerned that their children would be unable to express or even identify their ‘true self’ because of the impact that the *parents themselves* would have by encouraging or forcing their child to use CE’s. Parents worried that their child would be stunted in various ways if they sent their child the message that they must use CE’s in order to be loved, happy, successful, or complete. This provides greater insight into the issue of identity—it may not only be impacted by the neurobiological mechanisms that could be targeted by CE’s, but also by the interpretation and internalization of a message that you ought to be improved through CE.

There were a number of perspectives that differed between the group of parents with cognitively disabled children and the group of parents with nondisabled children. It is important to note, however, that there were far more similarities between the groups than there were differences; the central themes were discussed across participants. That being said, parents of cognitively disabled parents did provide a unique perspective in a number of ways. Firstly, these parents often had first-hand experience with products that could be used for CE (Ritalin, for example). They were able to offer insights of the benefits and consequences of various CE products based on their child (rather than a hypothetical situation). Additionally, based on their child’s experiences, they were able to express the difficulty they faced as parents to see their child suffer. They expressed that the way society was structured often worked against their child because of their ability differences, and as a result, sometimes they felt desperate and used approaches that they may not have even considered previously. Parents of nondisabled children often qualified their statements about CE’s by saying that they had never been in a situation where their child had faced issues with cognitive ability. Simply put, the primary difference was that parents of cognitively disabled children often had lived experience to draw from in informing their perceptions surrounding CE’s, whereas parents of nondisabled children were forced to rely on hypotheticals.

### The Meaning of Cognitive Enhancement

This study highlights important considerations pertaining to the definition of enhancement. Our definition of cognitive enhancement used for this study—a product or technology used to raise cognitive abilities *above* a normal level—was not always pertinent for most parents in this study. Parents constantly returned to the use of CE’s as a way to compensate for *disability*, despite this definition. Even parents that expressed some interest in CE products for children without a diagnosed disability often related their interest back to the enhancement somehow mitigating a struggle their child had or for compensating for another deficiency. Parents felt that using CE’s

to bring their child above a normal level of cognition would be motivated by competitiveness (stemming from the parent *or* child) or from a desire to better themselves (from the child’s perspective).

If strictly adhering to our initial definition of CE, concerns about using medication to reduce struggle would not have been acknowledged because those instances would not qualify as being intended to bring the user to an above-normal state (if we are examining above-normal based on that particular point in time). This is problematic given that parents envisioned that they may use CE products if they felt that their child’s struggle was making a significant negative impact on their lives, perhaps even in the absence of diagnosed disability. A common alternate definition of CE—the use of ‘internal methods’ (most often pharmaceuticals) to improve cognitive abilities in the absence of disease or disability—would not have captured all relevant issues either [5, 6]. Though this definition would encompass parents using certain CE products for struggles outside of disability and disease, it ignores the issue that conditions for cognition and disability are constantly changing, with CE use so that those who do not use would fall behind and could be considered disabled, even though their abilities would have been categorized as normal in the past. Though parents did not see this as an immediate threat for their own children, they acknowledged that if ability norms were to shift, it would be a major concern and would promote greater personal exploration of CE products.

It would appear that neither of the definitions currently employed in the CE literature are sufficient in encompassing all of the relevant issues pertaining to CE. If we are unconcerned about the use of CE’s to compensate for subclinical deficiencies or struggles, then the definition used at the outset of this study may be enough. In using this categorization, however, we may be overlooking important forces that are pushing individuals to feel the need to use these products for their struggles or more minor deficiencies. Based on the data from this study, these forces could relate to the stigma in our society of being different or ‘abnormal’ or gaps in our educational and health care systems that leave individuals feeling that they have no choice but to use CE products. If our primary concern is simply that individuals are using CE products without medical diagnosis, the second definition of CE is sufficient. This definition may lead us to under- explore issues of medicalization, increasing rates of diagnosis for various disabilities and mental illnesses, or the ramifications of using these products, even when someone has met the criteria for a diagnosis. The question that ultimately arises is this: do we feel that each of these issues are important enough to require exploration and action? If we as a society decide that indeed, all of these problems are meaningful to us, a more inclusive definition of what CE actually means is needed in order to address each of the problems that have been listed here.

### Implications

The perspectives that participants shared have a number of implications. Though further research will be required before changes could be undertaken, this study provides important data with respect to the development, uptake and regulation of CE’s. Parents from both groups identified that enhancements would be more acceptable if their child had a cognitive disability, was struggling or wanted CE’s while understanding the ramifications, and if the CE’s were safe as well as effective. Alternatively, CE’s were less desirable if they threatened feelings of acceptance, carried risk of physical harm, were used in the absence of medical need, and if it was stigmatized.

As discussed elsewhere in this paper, participants of this study spoke about the use of potential CE products for individuals with disease or disability. While this was a common topic for parents, we feel that CE in the absence of disability was still adequately addressed. Parents were generally against the use of natural products, surgery, pharmaceuticals or removable devices for the sole purpose of boosting in cognition in ‘healthy’ or non disabled individuals, for various reasons. They believed that CE could damage a child’s self-esteem, run the risk of putting the child’s physical health in jeopardy, and/or perpetuate unhealthy levels of cognitive competition between children. If CE’s were shown to be safe, parents were more open to them in cases where using them would help their child to feel that they ‘fit in’, but they still expressed that they would want to discuss alternatives (different teaching strategies, et cetera) to CE and only make the decision once the child was able to fully grasp the benefits and drawbacks of CE.

Struggling, failing to ‘fit in’, and falling behind were all concerning to parents. When presented with a scenario where their children were put at a disadvantage because other children were using CE’s, some of the parents acknowledged that they would be more interested in CE’s. Parents also acknowledged that if their children were struggling and CE’s would not harm them emotionally or physically, they would be more willing to consider CE use for their child. Therefore, while parents were generally hesitant about CE use, opinions may shift if CE products become more popular. Unsurprisingly, parents were averse to their children suffering or feeling that they were inadequate, so if the perceived level of what constitutes ‘normal’ rises, more parents may be considering CE’s for their children.

Parents viewed certain types of CE as more acceptable or desirable. Participants expressed greater aversion to enhancements that they perceived to be medical treatments. They discussed greater concern about the physical harm, stigma and the possibility of manipulating their child’s identity with such products. Natural products tended to be seen as safer and more a part of a regular routine or possible health-promoter. Based on these perspectives, it is possible that in the case of parents giving their children CE’s, products that are not marketed as treatment or being associated with medicine will be more popular because parents view them as being safer and socially acceptable. For this reason, it will be important to consider such products in future CE discourse, as these may be the most appealing to certain groups. Thus far, pharmaceuticals have dominated discussions about modes to achieve CE [9].

Parents discussed that *forcing* a child to use products in order to perform at an above-average level was unacceptable. Almost every participant expressed that they held a very negative view toward parents who were pressuring or forcing their children into using CE’s just for the sake of having greater cognitive skills. They viewed such parents as being too preoccupied with outward appearance, overly competitive, and failing to care properly for their child. The participants in this study viewed these parents as being the greatest concern for harm arising from CE use in children. Though representative data will be needed, this suggests that parents that push CE use for their children will be highly stigmatized by other parents.

Parents viewed CE use in children with limited autonomy as harmful and unacceptable. If this sentiment is true for the general population, we can expect that CE where children have little control (due to a lack of maturity or because parents are pressuring them) will be highly stigmatized for the parent. For this reason, a few parents expressed that they believed any CE use in children would be done in secret. This has important implications for potential regulation of CE’s. If parents are using CE’s for their children in secret, where will they be obtaining these products? Will these avenues of accessing CE be regulated to ensure safety, and will these distributors provide adequate information about the products? Though stigma may limit harm arising from CE use through discouraging individuals from practicing it, there is also potential that those who still choose to use CE’s may experience excessive harm because of that stigmatization by obtaining products through potentially unsafe sources [6]

The participants in this study tended to express that CE use should be monitored by a health care professional. Many parents saw this as a way to mitigate risk, as health practitioners would likely have greater knowledge of the risks and effectiveness of CE products. Some parents were uncomfortable with the idea of a broad-sweeping health policy to address CE use, but most agreed that the nature of CE’s necessitated some form of supervision from health professionals.

If the prevalence of CE is indeed a ‘common’ practice, as has been suggested by various academics and media outlets, health practitioners (physicians in particular) will need to be prepared to grapple with patient requests or questions regarding CE’s [4, 9]. Participants from this study seemed to expect that health professionals would be able to assess risk, need, and alternatives for CE products. Previous studies [13, 15] have identified that physicians and other health professionals may be uncomfortable dealing with CE’s due to risk, lack of knowledge, and what they perceive as their appropriate scope of practice. This issue may need addressing as parents view physicians as the best regulators of CE.

### Ethics

Ethics is to give guidance how technology ought to be used. However the parents in our study do not think in terms of what is ethical or unethical. Terms such as ethics, ethic or immoral were not mentioned once. The term moral was used twice by one parent of a child without a disabiliy in the context of it being a moral struggle for not being judgmental of parents using CE for their kids. This might indicate the limitation of reach of ethics discourses. Indeed it was speculated elsewhere [26] that people do not think in ethic or moral terms but in ability expectation and consequences terms. It also fits with a sentiment the eminent bioethicist Sherwin recently stated, “we [ethicists] lack the appropriate intellectual tools for promoting deep moral change in our society” [27]. Codes of Ethics are one instrument that used to outline the relationship between professionals and their clients. To just quote from three organizations; the Canadian Code of Ethics for rehabilitation professionals states, “Rehabilitation professionals are committed to facilitating the personal, social, and economic well-being of persons with a disability and/or disadvantage” [28]. Code of Ethics of the National Council of Rehabilitation Educators (NCRE) states, “the primary obligation of rehabilitation counselors is to clients, defined as individuals with or directly affected by a disability, functional limitation(s), or medical condition and who receive services from rehabilitation counselors” [29]. Code of Ethics of the Association of Professional Behavior Analysts (APBA) states: 2.10. “(b) Clients have a right to effective treatment (i.e., based on the research literature and adapted to the individual client)”; 2.10. “(c) Behavior analysts are responsible for review and appraisal of likely effects of all alternative treatments, including those provided by other disciplines and no intervention” [30]. Given the results of our study we submit that the quotes from the Code of Ethics of the three professional organizations strongly suggest that professionals have to be more involved in the discourse around CE.

According to the Canadian Code of Ethics for rehabilitation professionals, one task of the professionals is to facilitate “the personal, social, and economic well-being of persons with a disability and/or disadvantage” [28]. The interviews reveal that parents of children with a cognitive disability see CE as a possible threat to their well-being, with some acknowledging that it may lead to a rat race for ever increasing cognitive abilities that would leave their child even more behind and more negatively judged if they would not gain access to CE. Furthermore, if CE is indeed entering a stage of effectiveness and increased use, people so far seen as ‘cognitively able’ might be labeled as ‘cognitively impaired’, which would make them to compete with the people we perceive today as ‘cognitive impaired’ for ‘treatment’ resources. As to the Code of Ethics of the National Council of Rehabilitation Educators (NCRE), their professionals advise disabled people on the best way forward. In this case the question is whether they have to advise their clients to go for CE. Indeed one study showed that NCRE members believe that enhancements will come and that their clients very likely also want if not have to get enhancements down the road [31]. Studies exist that highlight that disabled people would not be content with a treatment to the norm if therapeutic enhancement (treatment that moves beyond the species-typical norm) would be available [32]. As to the code of Ethics of the Association of Professional Behavior Analysts (APBA), we must consider what is seen as effective treatment down the road. Various people anticipate that enhancements will be obtained by labelling them as health intervention and by labelling people without the enhancement as unhealthy [20]. Indeed some predict that health care consumers will drive the uptake of enhancements using the argument of choice [33]. This move will become even more pronounced if enhancement becomes a moral obligation as some ethicists propose (for some proposing the obligation see [33–37], for some contesting the obligation see [38, 39]). However, so far, of 1800 websites for medical and rehabilitation organizations in the USA only six mentioned CE one gave guidance on the topic and of the 203 Canadian organizations none covered the topic of CE [40]. We posit that most codes of ethics for medical and rehabilitation professionals are written with an understanding of terms such as treatment, health, rehabilitation and therapy that is benchmarked to the normal or species-typical body. However, increasingly therapeutic interventions have the potential to give recipients beyond species-typical body linked abilities (therapeutic enhancement [20]). Furthermore, non-therapeutic intervention leading to enhancements will add to a shift in the ability expectation landscape of the body including cognition related ability expectations and the meaning of health [20]. As such, these changes might necessitate actions on behalf of the professionals that were not on the radar screen when the codes of ethics were written.

### Future Directions

We believe more qualitative data is still needed that covers various sectors linked to disabled people such as disability service organizations, special education teachers and siblings of people with disabilities. Furthermore larger-scale studies that are representative of the parent population are needed to see if these perspectives we report here are widespread. If most parents do view non-treatment oriented products as more acceptable as has been the case in this study, concerns about parents giving their children products for CE ought to focus on products that are viewed as being health- promoters (versus disease-relievers). Research could include the safety, effectiveness, emotional impact, and prevalence of these products for children. Participants in this study expressed that one of the greatest sources of harm for CE use in children would come from coercive parents. Future studies could target parents with the characteristics that participants thought were associated with CE coercion—though accessing such a population and limiting social desirability bias would admittedly be extremely difficult. Research could focus on how parents with these characteristics view CE’s (do they see them as safe, effective, and so forth) as well as whether or not these parents would actually consider giving their children CE’s that are currently available, or if the perception that these parents would use CE’s quite freely is erroneous.

Parents identified a number of factors that influenced uptake of CE’s. What made these themes complex was that they were not necessarily discrete and one factor could be at odds with another. For example, parents expressed extreme hesitation in allowing their child to suffer or struggle excessively. At the same time, parents expressed great aversion to doing anything that could make their child feel unloved or rejected for who they were. It was unclear which factors ultimately won out over another. Interesting research could be pursued exploring how important different motivations/deterrents are relative one another. An example of potential research would be giving participants different CE scenarios that threatened one of the values identified in this study while facilitating another. Participants could then rate how likely they would be to use the product in each scenario.

Finally, this project was small-scale in nature. There were only twelve participants and they all resided within metropolitan areas of Alberta. Additionally, there was only one male parent that participated in this study. There are many perspectives that remain to be explored— fathers, parents from different areas, parents with different socioeconomic status, parents with children who are older and have greater independence, larger samples that generalize to the population. Any of these differences could have a significant impact on how CE’s are viewed and whether or not parents will decide they are right for their child to use. This study has provided some context for conducting such research. Perhaps more importantly, the participants in this study have provided rich data that will hopefully serve to better our understanding of CE’s and how parents conceptualize them.
